# Supplementary material for: Drosophila Fezf coordinates laminar-specific connectivity through cell-intrinsic and cell-extrinsic mechanisms
Source: eLife. 2018 Mar 7;7:e33962. doi: 10.7554/eLife.33962 (PMC5854465; doi:10.7554/eLife.33962)
Supplement: Supplementary file 2. [file elife-33962-supp2.pdf]

Contents

Overview 2
Quality control 3
Differential expression 9

date: 20 February 2018

Overview

Differential expression report for Jing Peng (Jing_Peng@hms.harvard.edu) from Matthew Pecot's lab (matthew_pecot@hms.harvard.edu).

The aim of this project is to identify targets of the Fezf transcription factor.

In order to do that, a total of 10 samples' total RNA, after FACS, has been sequenced:

- 5 WT
- 5 Fezf KO

In this report we analyze the quality of the sequencing data and perform a differential expression analysis for the contrast WT vs Fezf-KO.

Contact Victor Barrera (vbarrera@hsph.harvard.edu) from the Harvard Chan Bioinformatics Core for additional details.

The most recent update of this document occurred: Tue Feb 20 15:08:25 2018.

Samples metadata

Tha samples have the following metadata and QC mapping values.

```
         Mapped_reads Mapped_paired_reads Quality.format Sequence.length
dFezf1-A     13317700                   0       standard           25-75
dFezf1-B     12350308                   0       standard           25-75
dFezf1-C      8617814                   0       standard           25-75
dFezf1-D      9108406                   0       standard           25-75
dFezf1-E      5610065                   0       standard           25-75
FRT40-A       5178109                   0       standard           25-75
FRT40-B      13746878                   0       standard           25-75
FRT40-C       4712946                   0       standard           25-75
FRT40-D       8327462                   0       standard           25-75
FRT40-E      12203720                   0       standard           25-75
         Total_reads X.GC Duplicates     Name
dFezf1-A    25161124   42          0 dFezf1-A
dFezf1-B    21377559   42          0 dFezf1-B
dFezf1-C    18536542   42          0 dFezf1-C
dFezf1-D    17845365   42          0 dFezf1-D
dFezf1-E    14959511   42          0 dFezf1-E
FRT40-A      9285887   42          0  FRT40-A
FRT40-B     23095626   41          0  FRT40-B
FRT40-C     14545980   44          0  FRT40-C
FRT40-D     21009087   43          0  FRT40-D
FRT40-E     25812261   42          0  FRT40-E
         Sequences.flagged.as.poor.quality Average_insert_size genotype
dFezf1-A                                 0                   0      mut
dFezf1-B                                 0                   0      mut
```

```
     dFezf1-C                                 0                   0      mut
     dFezf1-D                                 0                   0      mut
     dFezf1-E                                 0                   0      mut
     FRT40-A                                  0                   0       wt
     FRT40-B                                  0                   0       wt
     FRT40-C                                  0                   0       wt
     FRT40-D                                  0                   0       wt
     FRT40-E                                  0                   0       wt
```

Quality control

Total reads

The number of total reads in the samples ranges from 9.2 million to 25.8 million and is highly variable. This is not surprising as the initial input for RNAseq was low given the complexity of obtaining samples.

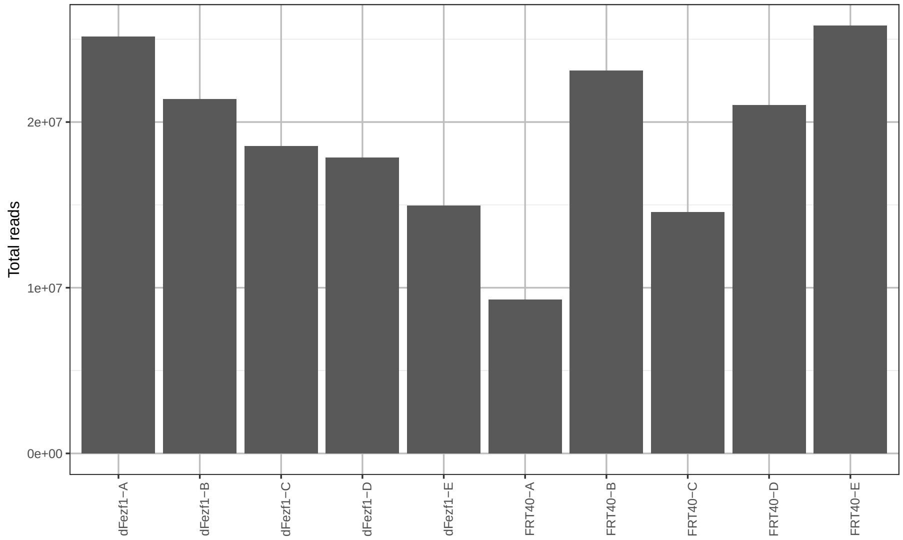

Mapped reads

We observe a similar distribution to the one observed for total reads. However, the decrease in values compared to total reads indicates low mappability.

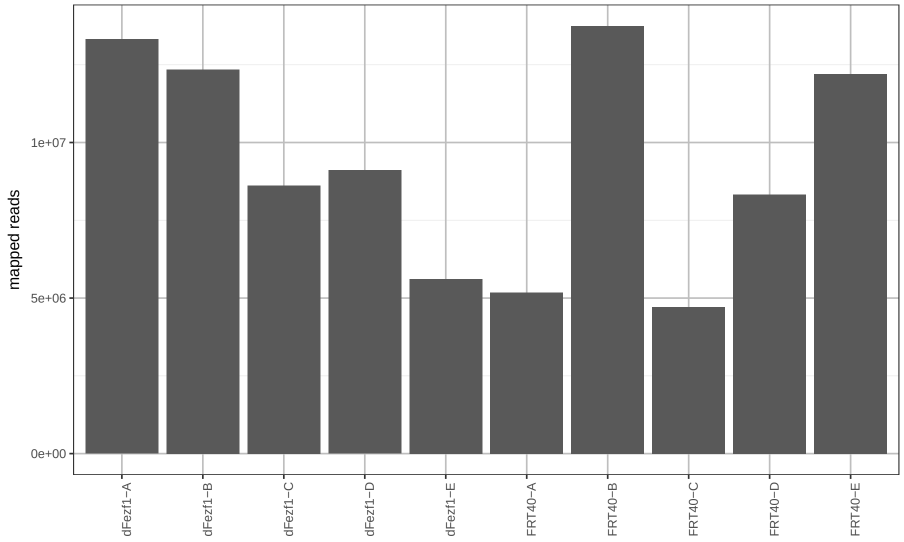

Mapping percentage

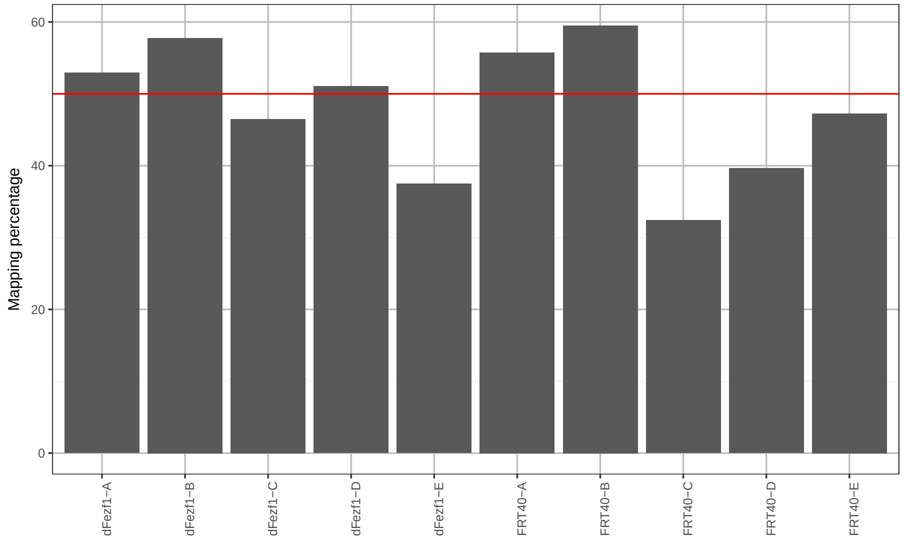

We observe low mapping percentages across all samples, and a disparity in the number of reads obtained between samples and mapping percentages. This was observed in a previous experiment and could indicate a problem with the normalization before loading the samples.

The sequencing facility report also showed a large proportion of undetermined sequences. This usually indicates a problem with the barcoding; however, examination of the most observed sequences in those files did not show an overrepresented barcode.

The samples presented with adapter contamination but removal did not increase the mapping rate.

Number of genes detected

We detect an average of 7,000 genes, which is half the total number of genes in Drosophila. As with other organisms, it is normal to have around half of the genome expressed for a specific cell type.

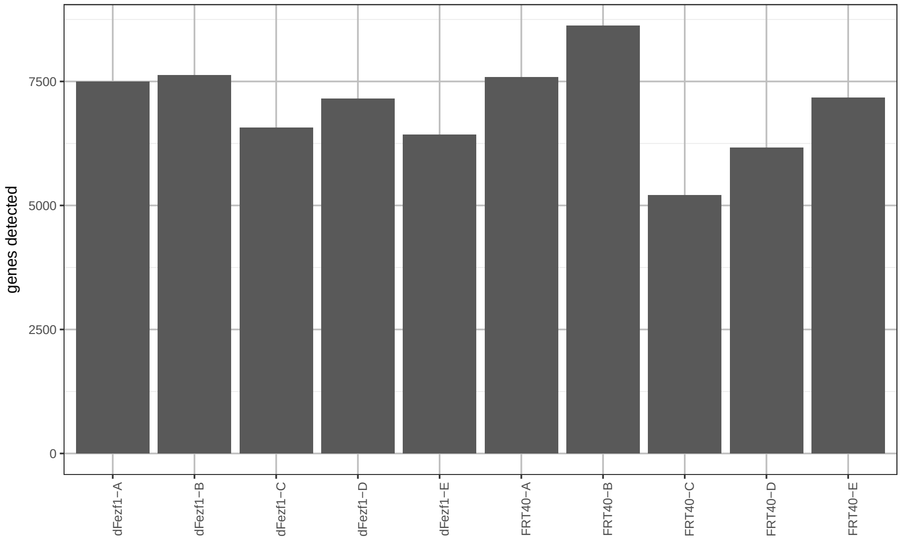

Boxplot of log10 counts per gene

We represent the distribution of counts for all genes for all the samples using boxplots to show the quartile values.

We observe that the samples have similar distributions, indicating that standard normalization procedures can be applied.

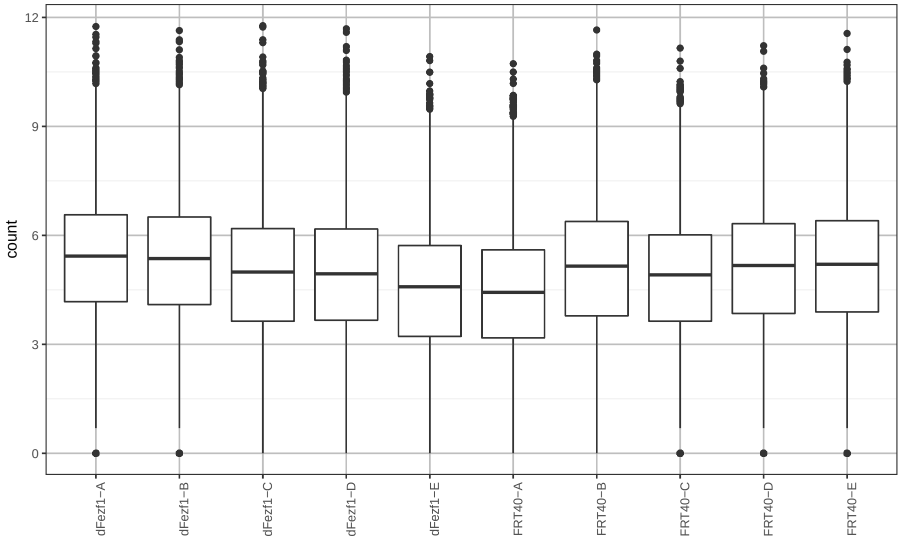

Correlation (Spearman) heatmap of TMM-normalized counts

The correlation heatmap allows us to visualize how samples cluster based on their expression profiles. We used Spearman correlation values to generate the heatmap.

We observe that samples cluster based on genotype. The WT sample FRT40-C has a lower correlation with other WT samples. This sample has the lowest mapping and gene detection rates.

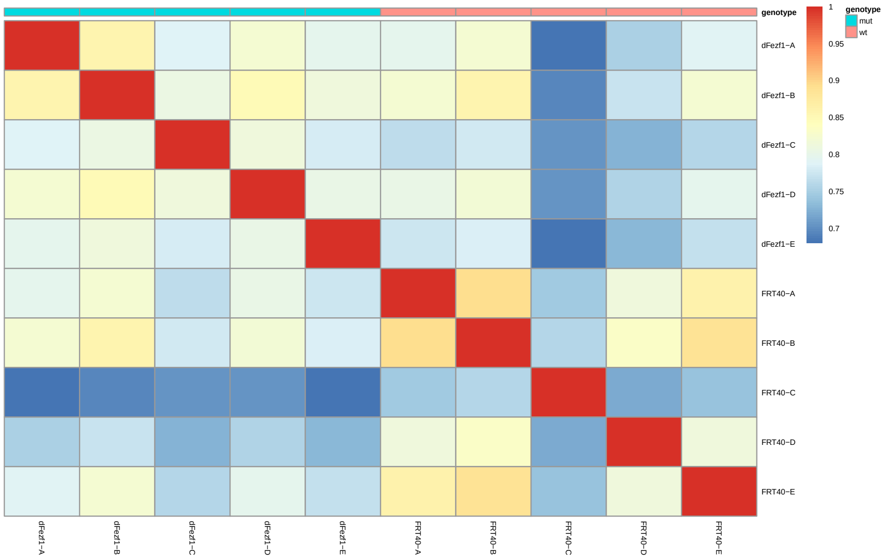

PCA plots

A PCA (Principal Component Analysis) performs a transformation over the data to obtain orthogonal vectors in such a way that the first principal component has the largest possible variance (that is, accounts for as much of the variability in the data as possible), and each succeeding component in turn has the highest variance possible under the constraint that it is orthogonal to the preceding components. PCA is often used to visualize distances and relatedness among samples.

We can observe that the samples separate clearly based on genotype. Although the FRT40-C sample is separated from the rest of the FRT40 samples, the differences in gene expression between the WT and the KO are much greater.

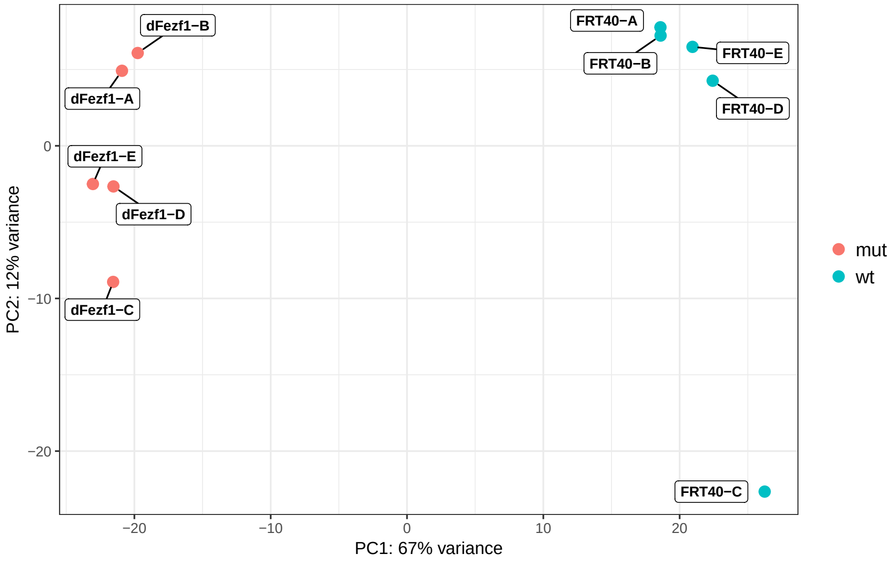

Differential expression

We perform the differential expression analysis for the contrast: KO(mutant) vs WT.

Plots

MA-plots

MA-plots are a great way to visualize the comparative expression metrics for a two group comparison. The x–axis is the average/mean expression over all the samples and the y-axis is the log2 fold change between the two conditions. The red dots represent the genes that are differentially expressed (adjusted pvalue <0.05).

We can observe a large number of differentially expressed genes.

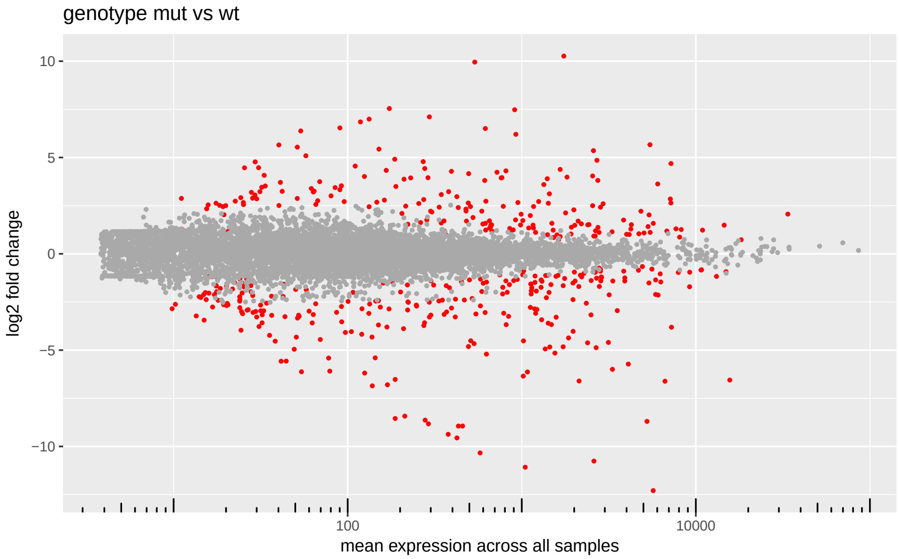

Volcano-plots

Volcano plots allow us to visualize statistical significance versus the magnitude of gene expression change. On the x-axis the log2 fold change between the two groups is represented. On the y-axis, the adjusted (for multiple testing) p-value for the test of Differential Expression. The green shade area surrounds genes with adj pval <0.05 and |logFC|>1.5.

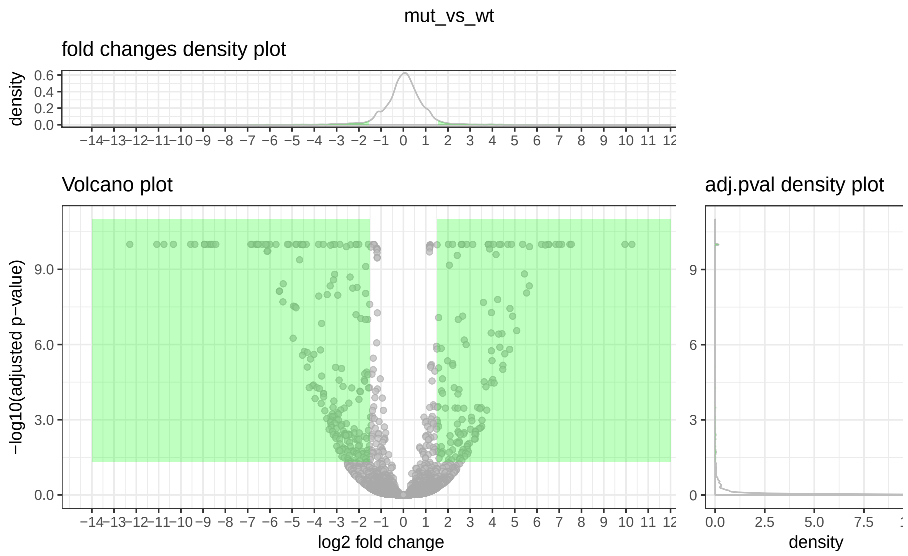


```
NULL
```

Top 12 significant genes expression

To sanity check the differential expression results, we plot the counts for the 12 top significant genes between the two conditions (mutant and WT). We observe clear differences in expression between the groups for the selected genes. The y-axis represent the counts in log2 scale.

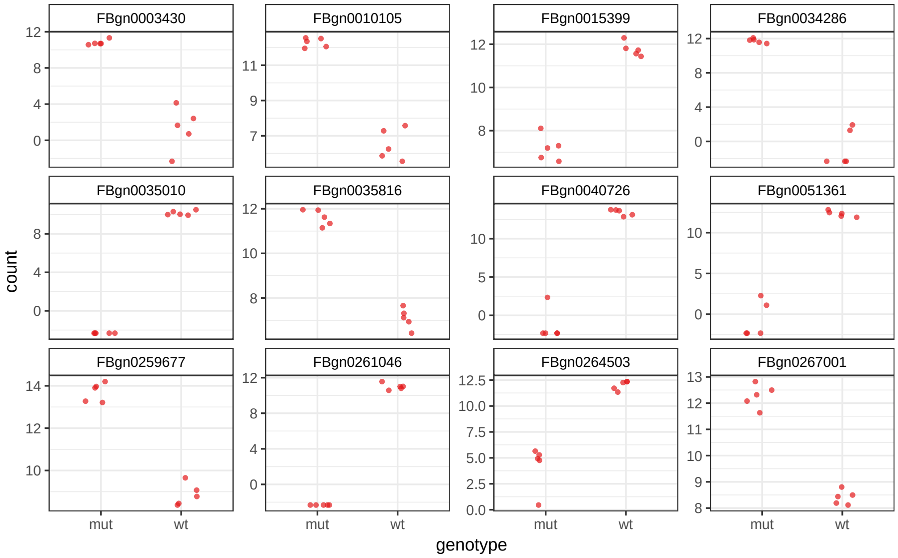

Differentially expressed genes

We provide a results file that contains the differential expression results for all the genes.

The file has 7 columns (+ number of samples) separated by tabs.

- id: Gene name.
- log2FoldChange: Effect size estimate in the form of log2 fold change. For the form A vs B (in the file name), it represents the log2 of the ratio [expression in condition A]/[expression in condition B]. A positive value indicates that the gene is upregulated in condition A compared to B. A negative one, downregulated.
- stat: Wald statistic value.
- pvalue: Wald test p-value.
- padj: BH adjusted p-values.
- symbol: gene symbol.
- description: gene description.
- Normalized rounded counts for each sample (multiple columns).

Transcription per million file

We generate and provide the file transcriptsPerMillion_Counts. It is a tab-separated file and it contains the transcript per million data for each sample.

The transcript per million concept is explained in the following http://www.rna-seqblog.com/rpkm-fpkm-and-tpm-clearly-explained/

Significant genes plots

Heatmap

We generated heatmaps for the comparison of WT vs KO using the expression values of significant genes. Each row represents the normalized expression of a significant gene and each column, a sample. This plot helps to identify patterns of expression of genes between samples in the form of clusters. Expression of genes is centered and scaled by row to highlight differences in each gene sample.

We can observe that samples clearly separate by the genotype.

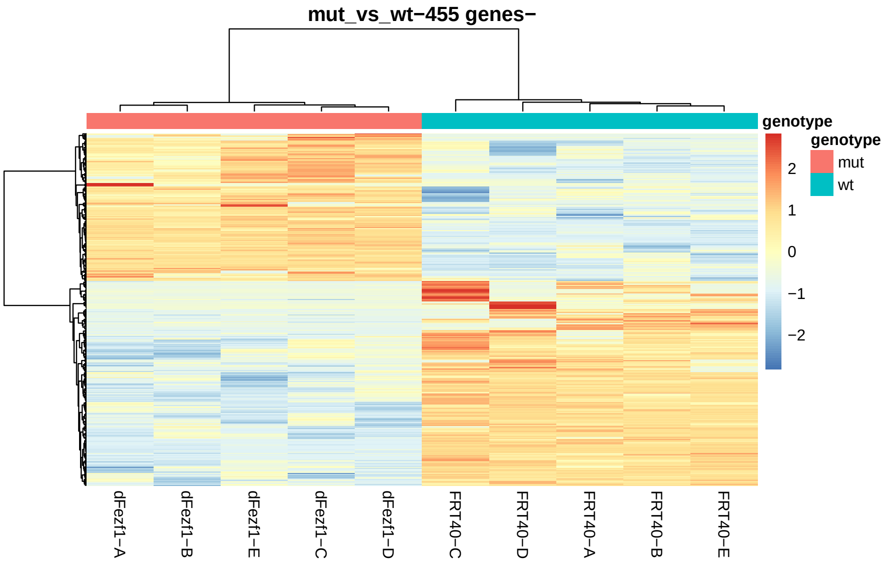

Heatmap-correlation (Spearman)

A heatmap plot of the correlation of expression patterns among samples. The samples clearly separate into the two groups.

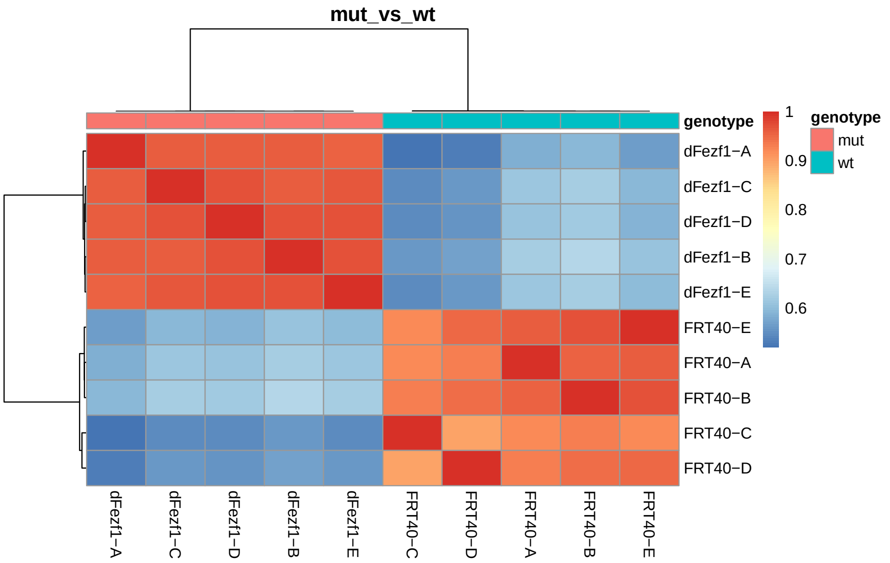

PCA with significant genes.

We performed a PCA using the information from only the significant genes. We can observe that the significant genes clearly separate the compared samples, with the first principal component representing 86% of all the variability.

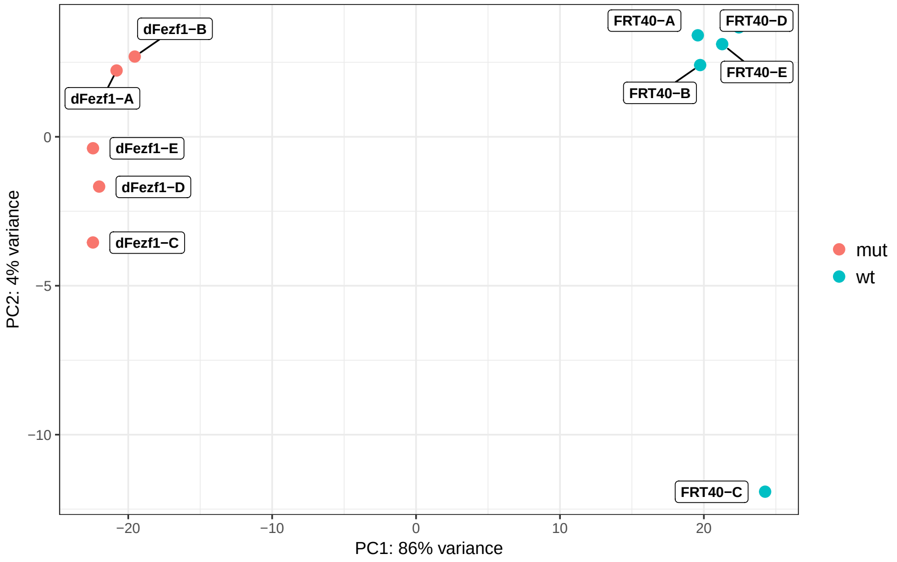

R session information

```
R version 3.4.3 (2017-11-30)
Platform: x86_64-apple-darwin15.6.0 (64-bit)
Running under: macOS High Sierra 10.13.1

Matrix products: default
BLAS: /System/Library/Frameworks/Accelerate.framework/Versions/A/Frameworks/vecLib.framework/Versions/A/libBl
LAPACK: /Library/Frameworks/R.framework/Versions/3.4/Resources/lib/libRlapack.dylib

locale:
[1] en_US.UTF-8/en_US.UTF-8/en_US.UTF-8/C/en_US.UTF-8/en_US.UTF-8

attached base packages:
 [1] stats4    parallel  grid      stats     graphics  grDevices utils
 [8] datasets  methods   base

other attached packages:
 [1] readr_1.1.1                AnnotationFilter_1.2.0
 [3] GenomicFeatures_1.30.0     AnnotationDbi_1.40.0
 [5] Biobase_2.38.0             GenomicRanges_1.30.1
 [7] GenomeInfoDb_1.14.0        IRanges_2.12.0
 [9] S4Vectors_0.16.0           BiocGenerics_0.24.0
[11] gridExtra_2.3              bindrcpp_0.2
[13] ggrepel_0.7.0              bcbioRNASeq_0.1.3
[15] customPlots_0.0.0.9000     logging_0.7-103
[17] dplyr_0.7.4                DEGreport_1.15.3
[19] quantreg_5.34              SparseM_1.77
[21] tximport_1.6.0             pheatmap_1.0.8
[23] CHBUtils_0.1               edgeR_3.20.7
[25] limma_3.34.6               gplots_3.0.1
[27] reshape_0.8.7              ggplot2_2.2.1
[29] BiocStyle_2.6.1            basejump_0.1.10
[31] DESeq2_1.18.1              SummarizedExperiment_1.8.1

loaded via a namespace (and not attached):
  [1] readxl_1.0.0                backports_1.1.2
  [3] circlize_0.4.3              Hmisc_4.1-1
  [5] AnnotationHub_2.10.1        plyr_1.8.4
  [7] ConsensusClusterPlus_1.42.0   lazyeval_0.2.1
  [9] splines_3.4.3               BiocParallel_1.12.0
 [11] digest_0.6.14               BiocInstaller_1.28.0
 [13] ensembldb_2.2.0             htmltools_0.3.6
 [15] viridis_0.4.1               gdata_2.18.0
 [17] magrittr_1.5                checkmate_1.8.5
 [19] memoise_1.1.0               cluster_2.0.6
 [21] ComplexHeatmap_1.17.1       Biostrings_2.46.0
 [23] annotate_1.56.1             Nozzle.R1_1.1-1
 [25] matrixStats_0.53.0          R.utils_2.6.0
 [27] prettyunits_1.0.2           colorspace_1.3-2
 [29] blob_1.1.0                  xfun_0.1
```

```
 [31] RCurl_1.95-4.10              genefilter_1.60.0
 [33] bindr_0.1                    survival_2.41-3
 [35] glue_1.2.0                   gtable_0.2.0
 [37] zlibbioc_1.24.0              XVector_0.18.0
 [39] MatrixModels_0.4-1           GetoptLong_0.1.6
 [41] DelayedArray_0.4.1           shape_1.4.3
 [43] scales_0.5.0                 vsn_3.46.0
 [45] DBI_0.7                      Rcpp_0.12.15
 [47] viridisLite_0.2.0            xtable_1.8-2
 [49] progress_1.1.2               htmlTable_1.11.2
 [51] foreign_0.8-69               bit_1.1-12
 [53] preprocessCore_1.40.0        Formula_1.2-2
 [55] htmlwidgets_1.0              httr_1.3.1
 [57] RColorBrewer_1.1-2           acepack_1.4.1
 [59] pkgconfig_2.0.1              XML_3.98-1.9
 [61] R.methodsS3_1.7.1            nnet_7.3-12
 [63] locfit_1.5-9.1               labeling_0.3
 [65] rlang_0.1.6                  reshape2_1.4.3
 [67] munsell_0.4.3                cellranger_1.1.0
 [69] tools_3.4.3                  RSQLite_2.0
 [71] evaluate_0.10.1              stringr_1.2.0
 [73] yaml_2.1.16                  knitr_1.18
 [75] bit64_0.9-7                  caTools_1.17.1
 [77] purrr_0.2.4                  nlme_3.1-131
 [79] mime_0.5                     R.oo_1.21.0
 [81] grr_0.9.5                    biomaRt_2.34.2
 [83] compiler_3.4.3               rstudioapi_0.7
 [85] curl_3.1                     interactiveDisplayBase_1.16.0
 [87] affyio_1.48.0                tibble_1.4.2
 [89] geneplotter_1.56.0           stringi_1.1.6
 [91] lattice_0.20-35              ProtGenerics_1.10.0
 [93] Matrix_1.2-12                psych_1.7.8
 [95] pillar_1.1.0                 GlobalOptions_0.0.12
 [97] data.table_1.10.4-3          cowplot_0.9.2
 [99] bitops_1.0-6                 Matrix.utils_0.9.6
[101] httpuv_1.3.5                 rtracklayer_1.38.3
[103] affy_1.56.0                  R6_2.2.2
[105] latticeExtra_0.6-28          bookdown_0.6
[107] RMySQL_0.10.13               KernSmooth_2.23-15
[109] codetools_0.2-15             gtools_3.5.0
[111] assertthat_0.2.0             rprojroot_1.3-2
[113] rjson_0.2.15                 GenomicAlignments_1.14.1
[115] Rsamtools_1.30.0             mnormt_1.5-5
[117] GenomeInfoDbData_1.0.0       hms_0.4.1
[119] rpart_4.1-12                 tidyr_0.7.2
[121] rmarkdown_1.8                dendsort_0.3.3
[123] shiny_1.0.5                  base64enc_0.1-3
```
